# Pectin as an Extraordinary Natural Kinetic Hydrate Inhibitor

**DOI:** 10.1038/srep23220

**Published:** 2016-03-21

**Authors:** Shurui Xu, Shuanshi Fan, Songtian Fang, Xuemei Lang, Yanhong Wang, Jun Chen

**Affiliations:** 1 Key Lab of Enhanced Heat Transfer and Energy Conservation, Ministry Education, School of Chemistry and Chemical Engineering, South China University of Technology, Guangzhou 510640, China

## Abstract

Pectin as a novel natural kinetic hydrate inhibitor, expected to be eco-friendly and sufficiently biodegradable, was studied in this paper. The novel crystal growth inhibition (CGI) and standard induction time methods were used to evaluate its effect as hydrate inhibitor. It could successfully inhibit methane hydrate formation at subcooling temperature up to 12.5 °C and dramatically slowed the hydrate crystal growth. The dosage of pectin decreased by 66% and effective time extended 10 times than typical kinetic inhibitor. Besides, its maximum growth rate was no more than 2.0%/h, which was far less than 5.5%/h of growth rate for PVCap at the same dosage. The most prominent feature was that it totally inhibited methane hydrate crystal rapid growth when hydrate crystalline occurred. Moreover, in terms of typical natural inhibitors, the inhibition activity of pectin increased 10.0-fold in induction time and 2.5-fold in subcooling temperature. The extraordinary inhibition activity is closely related to its hydrogen bonding interaction with water molecules and the hydrophilic structure. Finally, the biodegradability and economical efficiency of pectin were also taken into consideration. The results showed the biodegradability improved 75.0% and the cost reduced by more than 73.3% compared to typical commercial kinetic inhibitors.

Pectin, a kind of polysaccharides, widely exists in ripe fruits and certain vegetables. As possessing eco-friendly biodegradable natural product, pectin is widely used in medicine and food industry. Recently, we found another important application of pectin in oil and gas industry. It can solve one of the most vexing problems of gas industry, which are hydrate blockages during transportation in pipeline. Natural gas hydrate, an ice-like crystalline compounds formed by natural gas molecules and water molecules at high pressure and relatively low temperature^1^. Gas hydrate formation in pipelines will lead to pipeline plugged, stopping production. In extreme cases, it will result in abandonment and replacement of well^2^,^3^ and bring great potential risks to the oil and gas industry in flow assurance area. A serious hydrate plugs occurred at the leaking well on May 7 during accident of the BP Deep Water Horizon Oil Spill in 2010 when methane gas bubbles come in contact with cold seawater. Then, the cofferdam filled with hydrates, gas and oil and lost control. Until June 3, methanol injection was started and hydrate plugging was avoided^4^. Based on the events of the Deep Water Horizon disaster, hydrates blockages in any future containment operation have been emphasized highly. In order to avoid this kind of large scale gas hydrate formation, the petrochemical industry is working towards chemistries and dedicates to research inhibitors to avoid hydrate blockages^3^.

Broadly, hydrate inhibitors included conventional thermodynamic inhibitors (THIs) and novel low-dosage hydrate inhibitors (LDHIs). Application of THIs was becoming cumbersome due to high volumes required, causing significant financial implications and environmental issue, especially in offshore applications^5^,^6^. As a result, kinetic hydrate inhibitors (KHIs), a kind of LDHIs and typically injected 0.5 ~ 2.0 mass% of produced water, have been an alternative solution to prevent gas hydrate formation^7^. Nonetheless, most representative commercial KHIs, such as polymers of N-vinylcaprolactam (PVCap), N-methyl-N-vinylacetamide and 2-alkly-2-oxazoline^8^,^9^,^10^, have been limited because of their high-cost and insufficient biodegradation^11^,^12^. Herein, developing “cheap and green inhibitor” is a wise choice. One typical series of green inhibitors was Antifreeze proteins(AFPs)^13^, but it is costly and not easily obtainable. Another series were natural polymers such as tapioca starch^14^ and chitosan^15^, while its effectiveness was limited to 5.0 h at the subcooling temperature less than 5.0 °C.

Interestingly, as a kind of natural polymers, we found that pectin could retard methane hydrate formation and suppress the hydrate growth. Pectin is consists of linear regions of 1,4-linked-a-D-galacturonosyl units and their methyl esters^14^. Lots of oxygen or hydroxyl groups in the net structure of pectin may form hydrogen bonds with water molecules and perturb water structure, thus effectively inhibiting hydrate formation.

## Results and Discussion

### The inhibitory performance

**Figure 1:**
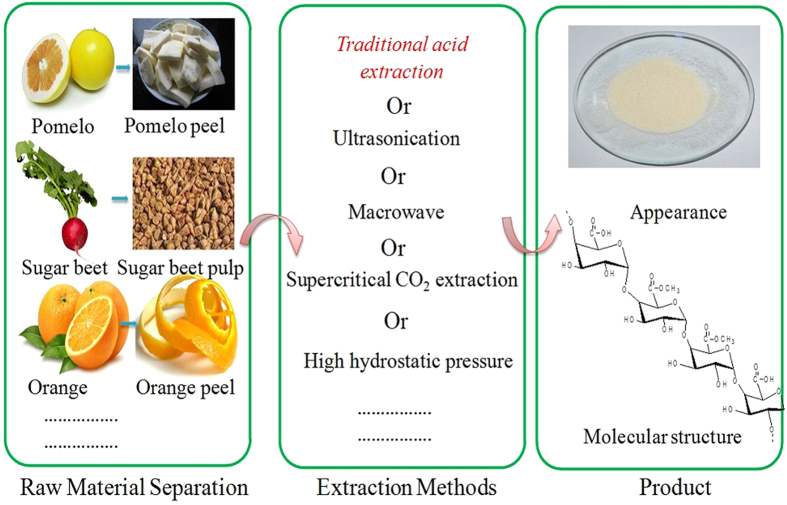
Simple extracted process and main chain structure of pectin. The traditional acid extraction method was used in this paper (These photographs of the figure were provided by Songtian Fang and this figure was created by shurui Xu).

As the structural unit of plant cells and the junction between the plant cell-wall, pectin can be easily extracted from citrus peel, apple pomace, orange peel, sugar beet pulp by various methods^15^. The process was shown in Fig. 1. We used traditional acid extraction extract pectin (Mw:264.624 KDa) from pomelo peel and investigated the inhibition effect of pectin on methane hydrate, and compared with the recognized effective KHI (PVCap, Mw:1.41 KDa) at the same conditions.

**Figure 2:**
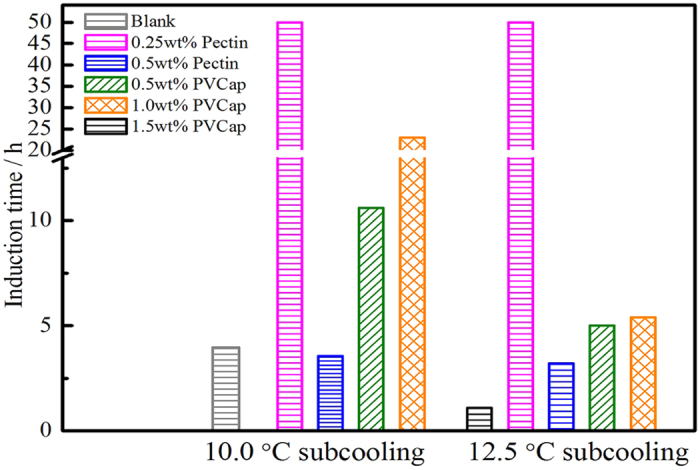
Onset time of methane hydrate formation in pure water system (the values in this figure are all based on 5 measurements).

**Figure 3:**
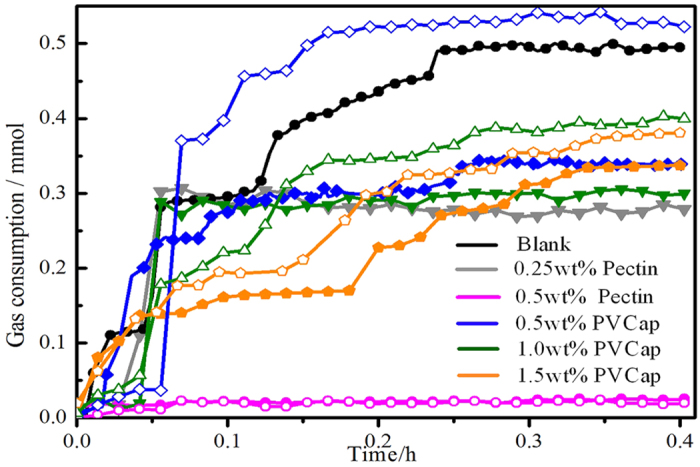
Gas consumption vs time profiles after initial hydrate formation in pure water system (the open and close symbol indicated 12.5 °C and 10.0 °C subcooling temperature, repectively).

As expected, the results proved that the pectin performed a powerful inhibition for methane hydrate formation. Figure 2 summarized the induction time of different concentrations of pectin and PVCap at two subcooling temperatures. Induction time was the time from the start cooling to the first detected hydrate formation and was the acknowledged evaluation parameter^16^,^17^, at which time hydrate had nucleated and started growing. Figure 3 displayed the gas consumption change with hydration time with the pectin or PVCap existence.

Compared with blank sample, both additives not only can prolong the induction time of methane hydrate but also can retard hydrate crystal growth. The inhibitory performance of PVCap was tested in the concentration range of 0.5 mass% to 1.5 mass%. The results showed that PVCap performed best at 1.5 mass%, which induction time is longest. When the methane hydrate appeared, it can keep a slow growth and the total gas consumption is less than that of experiment without additives. In the cases of pectin, it showed a much better inhibitory performance than PVCap. At 10.0 °C subcooling (9.5 MPa), the inhibitory performance of pectin at 0.25 mass% concentration was similar to PVCap at 0.5 mass%. While the concentration increased to 0.5 mass%, pectin showed a remarkable inhibitory performance and no hydrate was observed during the whole experiment process at two different subcooling temperatures. It is shown in detail in Figure S8. At 10.0 °C subcooling temperature, pectin showed extraordinary long inhibitory time and no gas consumed for methane hydrate formation. The induction time of pectin increased from 10.6 h to above 50.0 h, which surpassed 14.0 times than that of PVCap. Furthermore, pectin also showed powerful effect on inhibiting methane hydrate formation at relatively high subcooling temperature (subcooling 12.5 °C). Even the dosage of pectin was one-third of PVCap, pectin also can totally inhibit hydrate crystal growth in 50.0 h. The results proved that inhibitory performance of pectin outperformed PVCap and the injection dosage decreased by 66.0%. Compared with other typical natural inhibitors, the inhibitory performance of pectin was also outstanding. The tapioca starch as KHI was evaluated at subcooling 2.0 °C using methane (3.5 MPa). The results showed tapioca starch was able to delay the onset of hydrate formation by about 1.5 hours at 0.5 mass% concentration^18^. At about 4.5 °C subcooling and 4.5 MPa, the induction time of chitosan was no more than 150.0 minutes in methane system^15^. Besides, in the presence of AFPs, the average conversion ratio of water converted to hydrate had been more than 20% after 30 h in methane gas system (11.7 MPa and 12.0 °C subcooling)^19^. Above all, as a natural hydrate inhibitor, the inhibition activity of pectin to retard methane hydrate formation and reduce the crystal growth was efficient.

**Figure 4:**
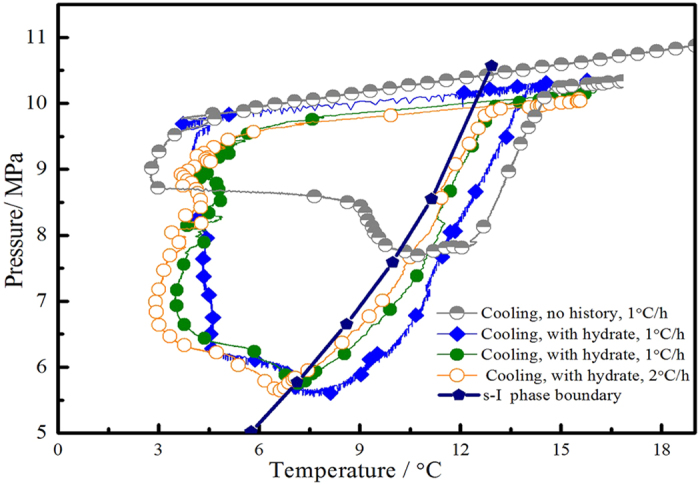
Pressure versus temperature plot showing example CGI method cooling/heating curves for methane-water system with 0.5 mass% pectin aqueous.

To further confirm the inhibitory of pectin on methane hydrate, crystal growth inhibition (CGI) method was used to determine crystal growth boundaries of pectin at 11.5 MPa. The CGI method, developed by Anderson *et al.* of Herriot Watt University^20^ and proved to be rapidly and reliably^7^,^21^ in practice. CGI method consisted of several hydrate growth–dissociation cycles. Following the first hydrate formation, a little measurable hydrate crystal (typically 0.5 mass% of aqueous phase as hydrate) was left and then the system was cooled back into the hydrate region to measure growth rates. Inhibition regions would be quantified according to the crystal growth patterns. By this means, all stochastic nucleation process was avoided, and hydrate growth rates only represented the ability of the KHI hindered crystal growth. Figure 4 showed cooling and heating curves for methane system with 0.5 mass% pectin aqueous plotted as pressure versus temperature. The s–I methane hydrate phase curve was shown for comparison^22^. On the first cooling run with no history (1.0 °C/h) nucleation eventually occurred after pressure and temperature conditions entered the hydrate region at 10.0 °C of subcooling. Then the cell temperature was heated rapidly to dissociate the hydrate present until only 0.74% the hydrate in cell.

Following the above, the temperature of the system was then cooled (1.0 °C/h) back into the hydrate region with a small fraction of hydrate present. No growth was observed until 4.9 °C of subcooling, at which point hydrate started growing at a very slow speed (0.02% hydrate/h). When the temperature of cell reached 3.6 °C (10.4 °C subcooling), the hydrate crystal growth rate suddenly increased from 0.45%/h to 0.78%/h. The maximum growth rate was no more than 2.2%/h although the temperature decreased to 2.0 °C, at which point the subcooling reached to 12.0 °C. Subsequent heating to the temperature outside the thermodynamic hydrate stability region for the system, the hydrate dissociation continued at a speed of 0.04%/h, which value is only 3% of decomposition rate for no-KHI system. As shown in Fig. 4, four hydrate growth–dissociation cycles were conducted to obtain reliable results. Thus from similar growth patterns, it apparently had four clear regions of KHI-induced growth/dissociation behavior: (1) a region where no growth/dissociation was observed, (2) a region where slow growth was observed, (3) a region where moderate growth was observed and (4) a region where slow dissociation was observed. These regions were the completed inhibition region (CIR), slow reduce growth region (RGR(S)), moderate reduce growth region (RGR (M))and slow dissociation regions (SDR)respectively. The boundary of CIR, RGR(S), RGR (M) and SDR were as follows: ΔT_s−I_ = −4.9 °C, ΔT_s−I_ = −10.4 °C, ΔT_s−I_ = −12.0 °C, ΔT_s−I_ = +1.5 °C.

**Figure 5:**
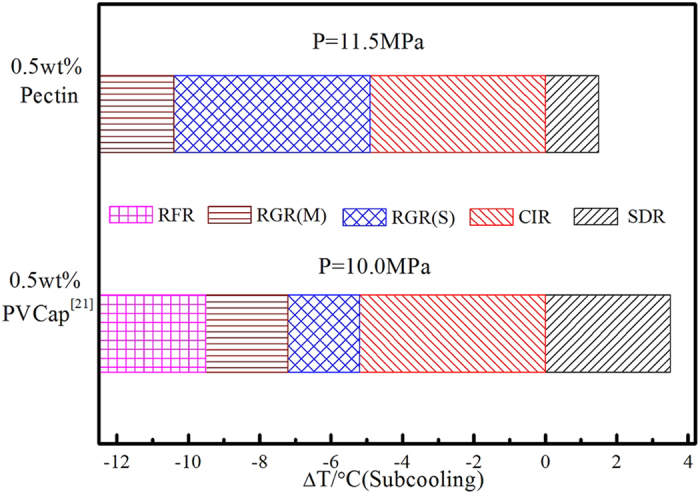
Experimentally determined subcooling extents of pectin-induced methane hydrate CGI regions relative to the s–I phase boundary.

Figure 5 summarized the subcooling extents of pectin-induced the CGI regions. Pectin could completely inhibit methane hydrate growth when the subcooling in the range of 4.9 °C and make the hydrate keep a relative slow growth trends when hydrate crystalline occurred. Moreover, it also successfully suppressed hydrate crystal rapid growth and the rapid failure region (RFR) was not observed even the subcooling extended to 12.0 °C, which was also accordance with induction time data. Compared with PVCap, the ability of the pectin to inhibit methane hydrate growth significantly improved. Although they possessed similar CIR boundary, the subcooling extents of pectin-induced slow reduce growth region and moderate reduce growth region extended more than 2.0 °C^21^. The maximum growth rate in RGR boundary (% water converted to hydrate per hour) was no more than 2.2%/h, which value was far less than 5.5%/h of growth rate for 0.5 mass% PVCap with methane^21^ (10.0 MPa). Under a very small fraction of hydrate crystal present (typically less than 0.15% water converted to hydrate), the crystal growth inhibition activity of pectin was better than commercial hydrate inhibitor, PVCap.

### Biodegradability and economy

In consideration of future large-scale application in the gas industry, especially in offshore application, novel inhibitors must meet environmental and biodegradability requirements. As a kind of natural materials, pectin broke through the poor biodegradability of KHIs. The biodegradability of pectin was evaluated based the BOD_5_/COD_cr_ ratio. Measurements of BOD_5_ and COD_cr_ were carried out based on HJ 505-2009 standards and GB/T 11914-1989 standards of china (shown in Table 1). It was well known that the BOD_5_/COD_cr_ ratio was commonly used to evaluate biodegrading property of wastewater and a higher BOD_5_/COD_cr_ ratio implied a higher degree of biodegradability^23^,^24^. The wastewater was usually considered as hardly biodegradable in the case of the ratio lower than 0.20^23^,^24^. From the above point, BOD_5_/COD_cr_ value of the PVcap aqueous was 0.259, which was not easy to be biodegraded and pectin possesses easily biodegrade nature. The biodegradability pectin aqueous was higher and improved almost 75.0% compared with PVCap. Moreover, since pectin exist in many kinds of fruit and vegetables and its content in citrus peel is more than 20.0%^25^ as well as its extract processing was mature, pectin is further cheaper than KHIs. Compared with using methanol ($0.33/kg), typically used at 35.0 mass% of produced water, pectin ($12.54/kg) provided a cost saving of approximately $121,792 per month at the intermediate water flow rate of 500BWPD^26^ and the cost was reduced by 45.7%. Besides, compared with the commercial KHIs ($23.52/kg), 0.5 mass% pectin can replace 1.0 mass% KHIs, which usage can save almost $397,834 per month^26^ in the same water flow rate and make the cost decreased by 73.3%.

### Inhibition mechanism

**Figure 6:**
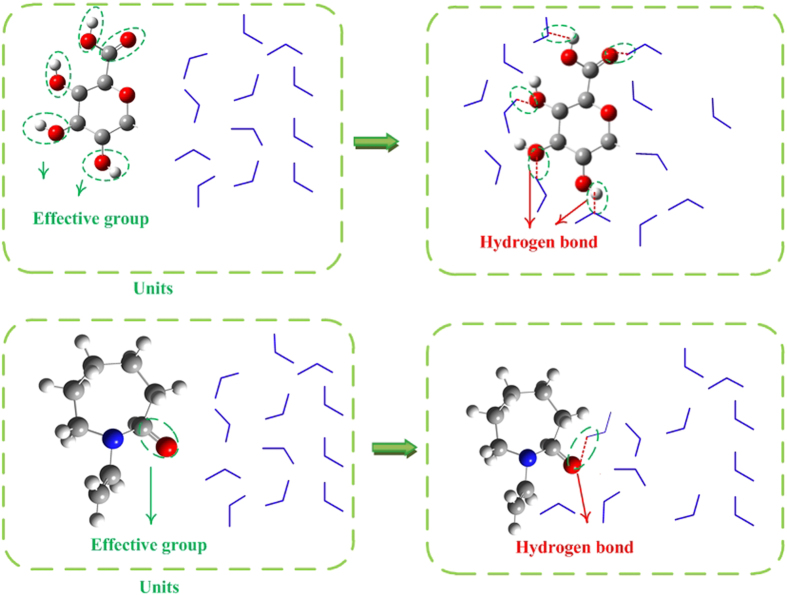
Schematic of active group of pectin and PVCap forming hydrogen bond with water molecules and perturbation of water molecules orders. Each monomer of pectin has five effective groups forming hydrogen bonds, while each monomer of PVCap has one effective group. Note that the graphic is intended for illustrative purposes and is not drawn to scale. (Red ball represent oxygen atom, blue ball represent nitrogen atom, gray ball represent carbon atom, white ball represent hydrogen atom, blue broken line represent water molecule, red dash line represent H-bond).

With lots of oxygen or hydroxyl groups in the pectin structure, pectin could form hydrogen bonds with water molecules, perturbing the water structure. The literatures^27^ indicated that water molecules could break the hydrogen bonds between hydroxyl and carbonyl groups of pectin through water and pectin molecular forming H-bonds. At same time, these effects result in the activity of water decreased^28^. Pectin as a novel hydrate inhibitor, its extraordinary inhibition activity was closely related to its hydrogen bonds between water molecules. According to previous mechanism of kinetic inhibition^29^,^30^,^31^, the oxygen atoms in carbonyl and hydroxyl groups of pectin (active group) can form hydrogen bonds with water molecules which will disrupt the water structure. This process hindered the water molecules to form hydrate cages and thus delayed the gas hydrate nucleation, as shown in Fig. 6. Furthermore, each monomer of pectin has five active groups. At the same mass, the active group amounts of pectin were about 2.6-fold of that of PVCap. That may be the reason why pectin has the better inhibitor performance than PVCap. Additionally, after nucleation, the hydrophilic structure enables pectin to retard hydrate crystal growth. Oxygen atoms of pectin bind to the surface of hydrate crystal through hydrogen bonds. Consequently, the a-D-galacturonosyl units of pectin can enhance the steric hindrance and thus suppress crystal growth^29^.

## Conclusion

In conclusion, as a kind of cheap natural material, pectin could tremendously improve induction time of methane hydrate nucleation and retard hydrate crystal growth. The induction time extended 10 times than the commercial hydrate inhibitor. Compared with other typical natural inhibitors, the inhibition activity of pectin increased 10.0-fold in induction time and 2.5-fold in subcooling temperature. Besides, it totally inhibited methane hydrate crystal rapid growth when hydrate crystalline occurred and the maximum growth rate was no more than 2.0%/h. Moreover, the biodegradability of pectin improved 75.0% and the cost reduced by more than 73.3%. With good inhibitory performance, high biodegradability and good economy, pectin is a good alternative KHI in gas and oil industry.

## Methods

Traditional acid extraction of pectin from pomelo peel was shown in supporting information and the detailed characteristics of pectin can be seen in Figure S2 and S3. Free radical polymerization of N-vinyl caprolactam was also shown in supporting information and the detailed characteristics of pectin can be seen in Figure S4 and S5. The experimental apparatus and procedure used to measure the induction time of pectin were the same as those used in our previous study^17^. And the definition of induction time can be seen in Figure S6. Besides, the novel crystal growth inhibition (CGI) method developed by Anderson *et al.* was also used in our experiment. The detailed experimental apparatus and procedures can be seen in supporting information. Finally, the test procedures of biodegradation were also performed.

## Additional Information

**How to cite this article**: Xu, S. *et al.* Pectin as an Extraordinary Natural Kinetic Hydrate Inhibitor. *Sci. Rep.* **6**, 23220; doi: 10.1038/srep23220 (2016).

## Supplementary Material

Supplementary Information

## Figures and Tables

**Table 1:** Biodegradation and residual of pectin and PVCap based the ratio of biochemical oxygen demand after 5 days (BOD_5_) and chemical oxygen demand (COD_cr_).

Samples	BOD_5_	COD_cr_	Degradation (%)	Residual (%)
Pectin	900.0	425.0	47.2	52.8
PVCap	1980.0	514.0	25.9	74.1
