# Author Correction: Pectin as an Extraordinary Natural Kinetic Hydrate Inhibitor

**DOI:** 10.1038/s41598-020-65654-1

**Published:** 2020-05-26

**Authors:** Shurui Xu, Shuanshi Fan, Songtian Fang, Xuemei Lang, Yanhong Wang, Jun Chen

**Affiliations:** Key Lab of Enhanced Heat Transfer and Energy Conservation, Ministry Education, School of Chemistry and Chemical Engineering, South China University of Technology, Guangzhou, 510640 China

Correction to: *Scientific Reports* 10.1038/srep23220, published online 21 March 2016

This Article contains an error in Figure 2. During the final preparation of the figures prior to publication, the wrong version of Figure 2 containing an incorrect graph legend was used. The correct Figure 2 appears below as Figure 1.

**Figure 1:**
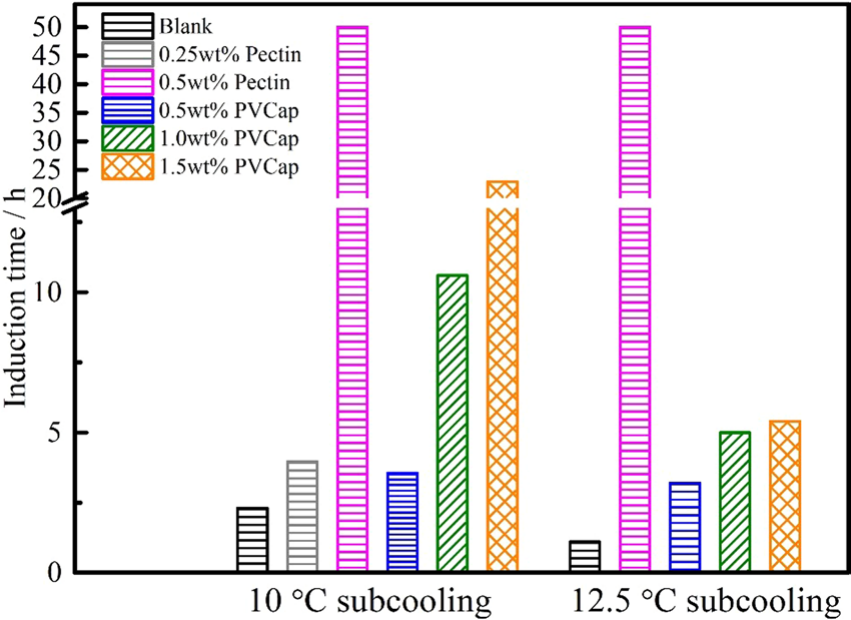
.

