# Supplementary material for: Pectin as an Extraordinary Natural Kinetic Hydrate Inhibitor
Source: Sci Rep. 2016 Mar 21;6:23220. doi: 10.1038/srep23220 (PMC4800399; doi:10.1038/srep23220)
Supplement: Supplementary Information [file srep23220-s1.pdf]

Electronic Supporting Information

“Pectin as an Extraordinary Natural Hydrate Inhibitor”

by Shurui Xu, Shuanshi Fan, Xuemei Lang, Yanhong Wang , Songtian Fang, Jun Chen,

Items:

- Supporting Methods and Materials

- Supporting Figure S8

Supporting Methods and Materials

Preparation of Pectin

Pectin was extracted using acid method as the preparation process is the following. Honey pomelo (Citrus grandis Osbeck) cultivated in Guanxi, Fujian province, was purchased from a local market of Guangzhou in November 2014. The collected peels of pomelo (ca.0.5 kg) were first chopped into pieces of 0.5 cm width and 0.5 cm length and steamed at 100 °C for 5 min to inactivate the enzymes and then was washed with deionized water three times at 50 °C. Next, citrus pectin is extracted in heated water at 95 °C from citrus peels at a pH of 2.00±0.05 for 45min and the ratio of the sample to water is 1:10. After discarding the pomelo peel by decompression filtration using buchner funnul, the filtrate contained pectin was obtained. Furthermore, pectin was coagulated with ethanol and precipitated out when absolute ethanol add to filtrate (ethanol: filtrate=2:1). After discarding the supernatant, the pectin was washed with ethanol four times and dried in vacuum oven at 50 °C until its weight had no any change. Finally, as shown in figure S1, the white sample was obtained (the yield of pectin was 2.5%) and stored at 4 °C.

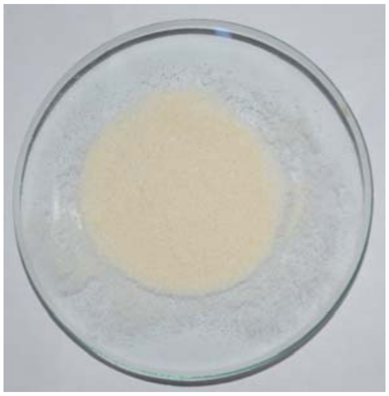

Figure S1 The appearance of citrus pectin was obtained in this study.

Characteristics of pectin

FTIR analysis was carried out over a NICOLET model NEXUS 670 spectrometer. Samples for the IR measurements were pressed into self-supporting wafers with a diameter of 2 cm in an IR-cell for investigation. The spectra were recorded from thin, lightly loaded KBr supported discs of the test samples at room temperature and the FTIR spectra of citrus pectin obtained in this study was shown in Figure S2.

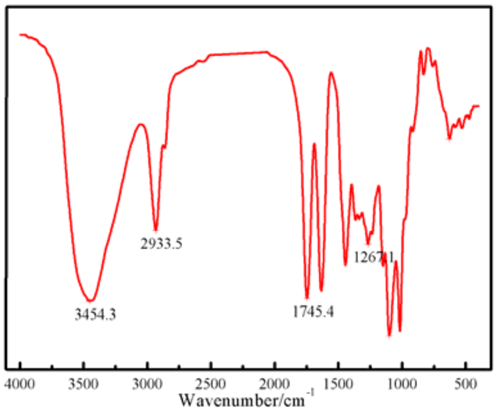

Figure S2 The FTIR spectra of citrus pectin obtained in this study in this study

Polymer number-average molecular weights (Mn) and molecular weight distribution (MWD = Mw/Mn) were measured by gel permeation chromatography (GPC) analyses carried out at 35°C and a flow rate of 0.06 mL/min, with 0.02 M KH_2PO_4 solution as the eluent, on a Water 1525 (Binary HPLC pump) GPC instrument equipped with refractive index detector (Water 2414).Chromatograms were processed with Breeze software (version 4.7). The results showed that Mw and MWD are equal to 264624 (shown in Figure S3) and 1.63, respectively.

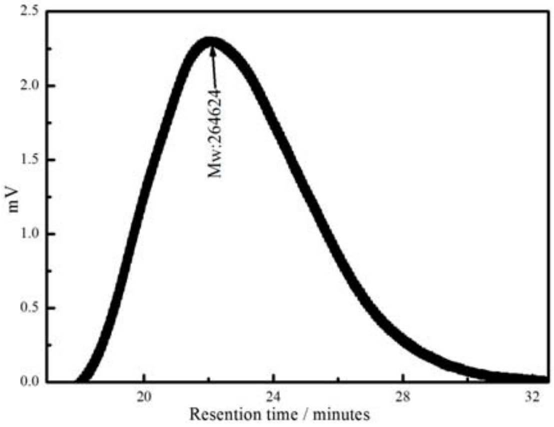

Figure S3 The plot of the Rid1A/ELU signal vs retention time in GPC tests.

Preparation of poly N-vinyl caprolactam (PVCap)

Homo-polymers of the N-vinyl caprolactam were synthesized using the following general procedure: The monomer was mixed with 1.0 wt % of the initiator 2, 2-azobis(2-methylpropionitrile) (AIBN) and 4 times its weight of ethanol in a Schlenk tube. The resulting solution was degassed on a high vacuum line and sealed under nitrogen. The reaction mixture was then stirred and allowed to polymerize at 75 °C for typically 12 h. Solvents were removed under reduced pressure to leave a white solid.

Characteristics of PVCap

FTIR analysis was carried out over a NICOLET model NEXUS 670 spectrometer. Samples for the IR measurements were pressed into self-supporting wafers with a diameter of 2 cm in an IR-cell for investigation. The spectra were recorded from thin,

lightly loaded KBr supported discs of the test samples at room temperature and the FTIR spectra of PVCap obtained in this study was shown in Figure S3. The peaks at 3106.52 cm^{-1}, 1629.02cm^{-1}, 1479.58cm^{-1}, and 1660.68cm^{-1}, corresponding for-CH, -C=C,-C-N and C=O of ester stretching and detailed information shown in figure S4.

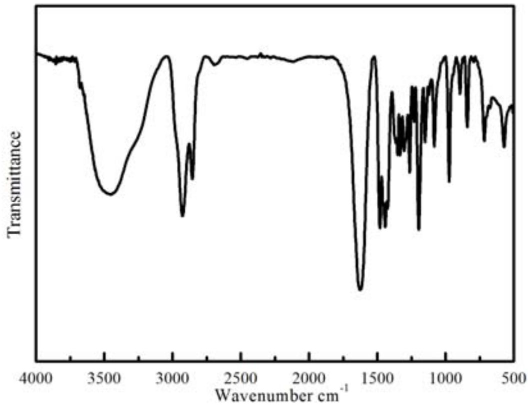

Figure S4 The FTIR spectra of Poly N-vinyl caprolactam obtained in this study in this study.

Polymer weight-average molecular weights (Mw) and molecular weight distribution (MWD = Mw/Mn) were measured by gel permeation chromatography. GPC analysis carried out at Agilent 1100 a flow rate of 1.0 mL/min with THF solution as the eluent. The results are shown in Figure S5 and the value of Mw and MWD are equal to 1590 and 1.10, respectively.

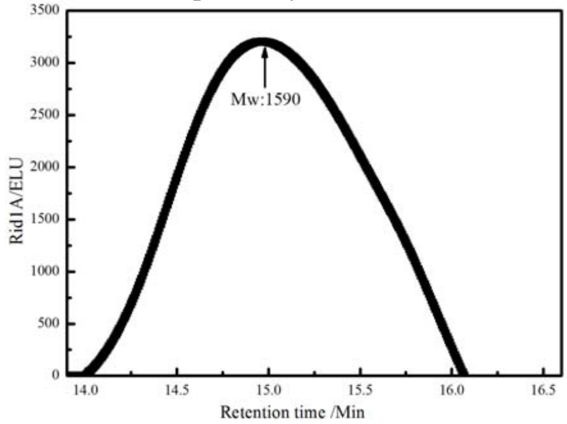

Figure S5 The plot of the Rid1A/ELU signal vs retention time in GPC tests.

Hydrate Inhibitor performance tests

1. Induction time tests

Experimental epuipment

The inhibitory performance of pectin in methane +water system was carried out in a low temperature test chamber (Xutemp Temptech Co. Ltd.), consisted of six identical magnetic-stirred steel cells with a volume of 100 cm^3. The isothermal cooling method was used to measure the onset time of pectin. The detailed test procedures are as follows: KHIs were dissolved in the distilled water to form a certain concentration solutions respectively. (2) 60 g prepared solutions was charged in to the cell (100 ml) by vacuum pump. (4) When the temperature of the cooling bath was

stable at 305K the cell was loaded with methane gas to the desired pressure and stirred at 700 rpm. (5) Cooling the cell to the desired temperature (0.5 °C) and keep 50 h. The hydrate onset time, and gas consumption in the presence of pectin from the measurements of temperature and pressure changes during hydrate formation are included. Additionally, every sample was tested 5 times at same conditions to avoid stochastic.

The definition of induction time

The induction time (t_i) is defined as the time from the start of cooling to the first detectable hydrate formation. Figure S6 displayed typical curves of pressure and temperature versus time recorded in an experimental run (1.0 wt% PVCap solutions at 10.0 °C subcooling). As showed in Figure S6, at the beginning of cooling process, the experimental pressure drops with the temperature drops in a close system, and the pressure dropped during the stable period of P and T indicated hydrate began forming in the cell. Besides, Hydrate formation is an exothermic process which can be seen by the spike in temperature at the beginning of the growth period, which also could be seen in Figure S6. The analysis t_i was displayed in Figure S6.

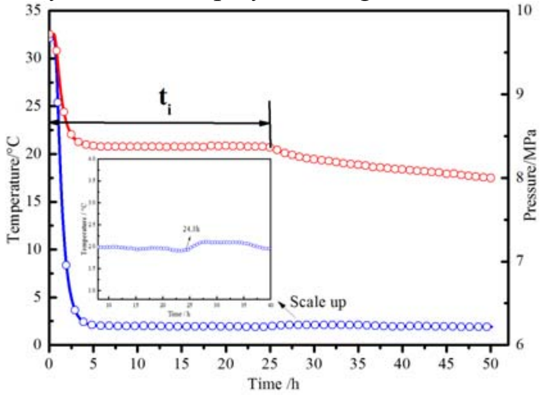

Figure S6. Data from the isothermal cooling test with 1.0wt% PVCap at 10.0 °C sub-cooling.

2. Hydrate crystal growth inhibition regions tests

Background to the CGI method

In order to overcome the stochasticity of the nucleation step, a crystal growth inhibition (CGI) method was developed by Anderson et al[1], which method is an evolution of the "second germination" technique[2]. According to the CGI method, the hydrate crystal inhibition regions can be divided to four parts and they are complete inhibition region (CIR), reduced growth rate region (RGR), rapid failure region (RFR) and slow dissociation rate region (SDR). The detailed classification based on the amount of aqueous phase converted to hydrate crystal was shown in Table S1.

Table S1 KHI Induced CGI region nomenclature and typical hydrate growth Rates

CGI region		Typical growth rates V_g(%water/hr)	Growth rate description
CIR		0.00	No Growth
RGR	(VS)	0.01 (V_g<0.05)	Very slow
	(S)	0.1 (0.05≦V_g<0.5)	Slow
	(M)	1 (0.5≦V_g<5)	Medium
RFR		10 (5≦V_g)	Rapid
SDR		Dissociation rate one order of magnitude less than for no KHI	(Abnormally) slow dissociation

Experimental Equipment

Tests were conducted on in-house designed 334ml volume high pressure stainless steel, as shown in Figure S7. The allowable operational temperature and pressure ranges for the vessel were -50 to 250 °C and 0 to 30.0 MPa, respectively. The temperature of the vessel was controlled by a thermostatted bath (Huber CC805). High-pressure nitrogen and vacuum pump were used to convey solutions. In both apparatus, two platinum resistance thermometers (Westzh WZ-PT100) with an accuracy of ± 0.1 °C were placed inside vessels to measure the temperatures in the vapor and liquid phases. A pressure transducer (Senex DG1300-ZN-C2-25/CD5) with an accuracy of ± 0.01 MPa was used to measure the internal pressure of vessels. The experimental data were recorded by a data logger (Agilent 34970A).

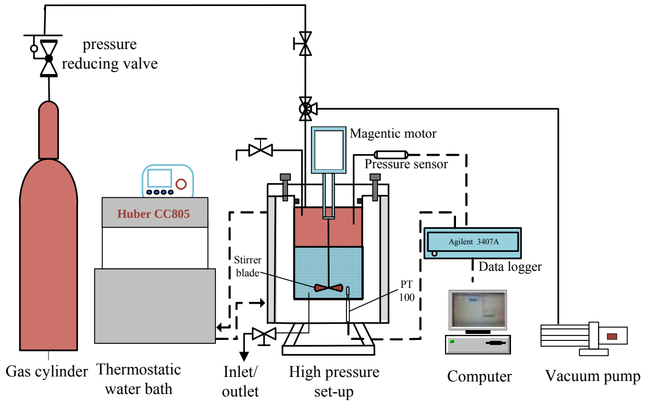

Figure S7. Schematic illustration of the 334 ml high pressure (max 300 bar) autoclave cells used in experiments.

The CGI experimental procedure

The CGI method consists of a number of hydrate growth-dissociation cycles, leaving a small but measurable hydrate seed (typically, <1.0% of aqueous phase as hydrate) following the first growth cycle, ahead of cooling back into the hydrate

region to observe growth patterns and use these to quantify inhibition regions. The general experimental procedures are as follows:

(1) Following gas charging to target pressure at a temperature outside the hydrate region, the system was first cooled rapidly to a certain high subcooling temperature to induce hydrate formation.

(2) Following initial rapid hydrate formation, the system was then warmed in steps to dissociate most of the hydrate formed, leaving only a small fraction remaining (typically < 0.5% of water converted), while assessing the region of slow dissociation behavior.

(3) Cell temperature was then reduced again at a constant cooling rate (typically 1.0 °C / hr) to observe clear changes in growth rate as a function of subcooling temperature.

(4) Steps 2-3 were repeated a number of times to examine repeatability.

(5) Finally, following a repeat of Step 2, the system was step-cooled with a small fraction of hydrate present to confirm the extent of the complete inhibition and very slow growth regions where appropriate.

The tests of biodegradation

BOD indicates the elimination of an organic substance from water, and allows only indirect conclusions about biodegradation[3]. And BOD generally refers the consumption of dissolved oxygen in the process of decomposing the organic substance from water. The test procedures of BOD_5 were based on HJ 505-2009 standards of china. The general experimental procedures are as follow:

(i)The water samples contained pectin or PVCap filled with the sealed bottles of dissolved oxygen and the mass concentration of dissolved oxygen in water sample would be measured by dissolved oxygen meter (the model is SPX-250BS-II), which value was marked as $BOD_{b.}$

(ii) Then the samples would be cultivated 5 day ± 4 h at 20 °C in the dark.

(iii) The mass concentration of dissolved oxygen in water sample would be measured by the same meter after step ii and the value was marked as BOD_a.

(iv) The value of BOD_5 was equal to BOD_a minus $BOD_{b.}$

Additionally, the test procedures of chemical oxygen demand (COD_{cr}) were based on dichromate method according to GB/T 11914-1989 standards of china and the detailed process would not be described here.

The BOD_5/COD_{cr} ratio is a key parameter in ready biodegradability of water quality evaluation process[4]. In general, the biochemical process of wastewater would be easier when BOD_5/COD_{cr} ratio is higher. The degree of biodegradation can be split into four categories according to the BOD_5/COD_{cr} ratio[5]:

1. Easily biodegradable: $BOD_5/COD_{cr} > 0.45$
2. Biodegradable: $BOD_5/COD_{cr} > 0.3$
3. Difficultily biodegradable : $BOD_5/COD_{cr} < 0.3$
4. Hardly biodegradable :$BOD_5/COD_{cr} < 0.2$

Reference

(1) Ross Anderson, H. M., Bahman Tohidi. *Proceedings of the 7th International Conference on Gas Hydrates* **2011**.

(2) Duchateau, C.; Dicharry, C.; Peytavy, J.-L.; Glénat, P.; Pou, T.-E.; Hidalgo, M. In *Proceedings of the 6th International Conference on Gas hydrates, Vancouver, Canada* **2008**; Vol. 5582.

(3) Reuschenbach, P.; Pagga, U.; Strotmann, U. *Water Research* **2003**, *37*, 1571.

(4) Arslan-Alaton, I.; Akmehmet Balcioglu, I. *Archives of environmental contamination and toxicology* **2002**, *43*, 425.

(5) Wei, H. J. S. *Jiangsu Environmental Science and Technology* **2004**, *3*, 003.

Figure S8. The typical pressure and temperature plots obtained from the isothermal cooling test with 0.5wt% pectin. (a indicated 10.0 K sub-cooling and b indicated 12 sub-cooling).

In all experiment process, no hydrate was observed during the whole experiment process for system adding pectin with mass fractions of 0.5wt% under two different sub-cooling.

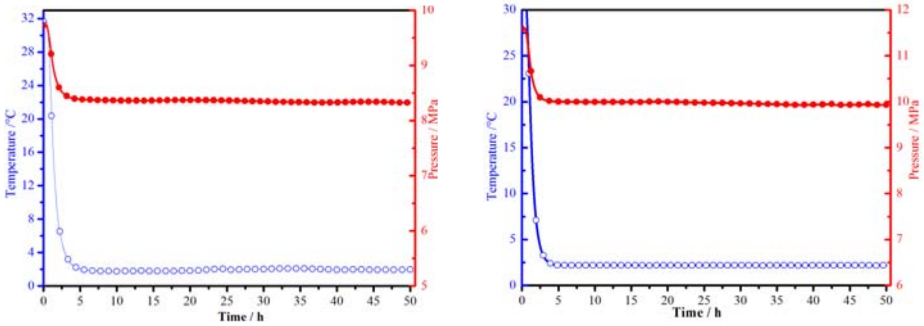

(a) (b)
